# *N*-(3,5-Dimethyl­phen­yl)-4-methyl­benzene­sulfonamide

**DOI:** 10.1107/S1600536809053057

**Published:** 2009-12-16

**Authors:** B. Thimme Gowda, Sabine Foro, P. G. Nirmala, Hartmut Fuess

**Affiliations:** a Department of Chemistry, Mangalore University, Mangalagangotri 574 199, Mangalore, India; b Institute of Materials Science, Darmstadt University of Technology, Petersenstrasse 23, D-64287 Darmstadt, Germany

## Abstract

In the title compound, C_15_H_17_NO_2_S, the dihedral angle between the two aromatic rings is 53.9 (1)°. The crystal structure features inversion-related dimers linked by N—H⋯O hydrogen bonds.

## Related literature

For preparation of the title compound, see: Shetty & Gowda (2005 ▶). For our study of the effects of substituents on the structures of *N*-(ar­yl)-aryl­sulfonamides, see: Gowda *et al.* (2010 ▶); Nirmala *et al.* (2009**a* ▶,b* ▶). For related structures, see: Gelbrich *et al.* (2007 ▶); Perlovich *et al.* (2006 ▶).
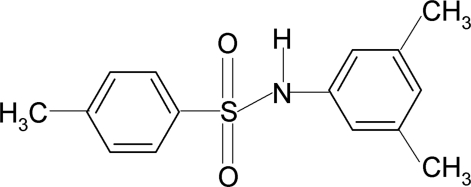


## Experimental

### 

#### Crystal data

C_15_H_17_NO_2_S
*M* *_r_* = 275.36
Triclinic, 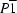

*a* = 8.311 (1) Å
*b* = 8.521 (1) Å
*c* = 11.412 (1) Å
α = 99.86 (1)°
β = 97.62 (1)°
γ = 108.14 (1)°
*V* = 741.60 (14) Å^3^
*Z* = 2
Mo *K*α radiation
μ = 0.22 mm^−1^
*T* = 299 K
0.45 × 0.45 × 0.40 mm

#### Data collection

Oxford Diffraction Xcalibur diffractometer with a Sapphire CCD detector
Absorption correction: multi-scan (*CrysAlis RED*; Oxford Diffraction, 2009 ▶) *T*_min_ = 0.909, *T*_max_ = 0.919
4897 measured reflections
3025 independent reflections
2525 reflections with *I* > 2σ(*I*)
*R*_int_ = 0.012

#### Refinement

*R*[*F* ^2^ > 2σ(*F* ^2^)] = 0.040
*wR*(*F* ^2^) = 0.118
*S* = 1.06
3025 reflections
179 parameters
1 restraint
H atoms treated by a mixture of independent and constrained refinement
Δρ_max_ = 0.27 e Å^−3^
Δρ_min_ = −0.31 e Å^−3^

### 

Data collection: *CrysAlis CCD* (Oxford Diffraction, 2009 ▶); cell refinement: *CrysAlis RED* (Oxford Diffraction, 2009 ▶); data reduction: *CrysAlis RED*; program(s) used to solve structure: *SHELXS97* (Sheldrick, 2008 ▶); program(s) used to refine structure: *SHELXL97* (Sheldrick, 2008 ▶); molecular graphics: *PLATON* (Spek, 2009 ▶); software used to prepare material for publication: *SHELXL97*.

## Supplementary Material

Crystal structure: contains datablocks I, global. DOI: 10.1107/S1600536809053057/bt5135sup1.cif

Structure factors: contains datablocks I. DOI: 10.1107/S1600536809053057/bt5135Isup2.hkl

Additional supplementary materials:  crystallographic information; 3D view; checkCIF report

## Figures and Tables

**Table 1:** Hydrogen-bond geometry (Å, °)

*D*—H⋯*A*	*D*—H	H⋯*A*	*D*⋯*A*	*D*—H⋯*A*
N1—H1*N*⋯O1^i^	0.83 (2)	2.11 (2)	2.933 (2)	170 (2)

Symmetry code: (i) 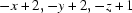.

**Fig. 1.**
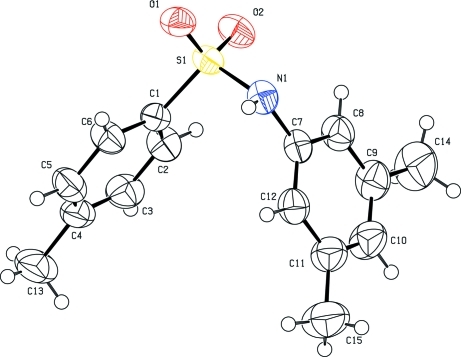
Molecular structure of the title compound, showing the atom labelling scheme and displacement ellipsoids are drawn at the 50% probability level.

**Fig. 2.**
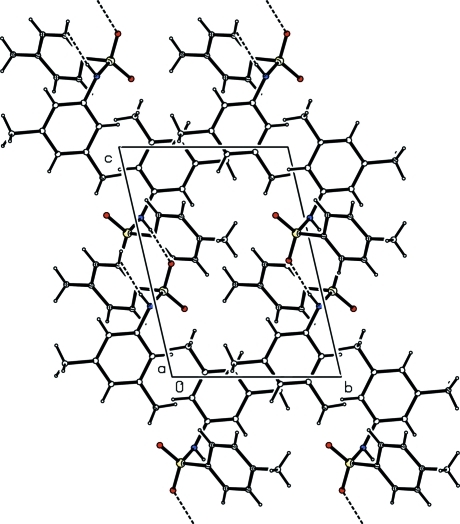
Molecular packing of the title compound with hydrogen bonding shown as dashed lines.

## supplementary crystallographic information

### Comment

In the present work, as part of a study of the effect of substituents on the crystal structures of *N*-(aryl)-arylsulfonamides (Gowda *et al.*, 2010; Nirmala *et al.*, 2009*a,b*), the structure of *N*-(3,5-dimethylphenyl)4-methylbenzenesulfonamide (I) has been determined. The conformation of the N—C bond in the C1—SO_2_—NH—C7 segment of the structure has *gauche* torsions with respect to the S═ O bonds (Fig. 1). Further, the conformation of the N—H bond is *anti* to one of the 3-methyl groups and *syn* to the other in the aniline benzene ring. The molecule is bent at the *S* atom with the C1—SO_2_—NH—C7 torsion angle of 56.8 (2)°, compared to the values of -51.6 (3)° in *N*-(phenyl)4-methylbenzenesulfonamide (II) (Gowda *et al.*, 2010), 56.7 (3)° in *N*-(3-methylphenyl)4-methylbenzenesulfonamide (III) (Nirmala *et al.*, 2009*a*), 67.9 (2)° in *N*-(3,5-dimethylphenyl)benzenesulfonamide(IV)(Nirmala *et al.*, 2009*b*) and -61.0 (2)° in *N*-(2,5-dimethylphenyl)4-methylbenzenesulfonamide (V) and -61.8 (2)° in *N*-(3,4-dimethylphenyl)4-methylbenzenesulfonamide (VI) (Gowda *et al.*, 2010).

The two benzene rings in (I) are tilted relative to each other by 53.9 (1)° compared to the values of 68.4 (1)° in (II), 83.9 (1)° in (III), 54.6 (1)° in (IV), 49.4 (1)° in (V) and 47.8 (1)° in (VI). The other bond parameters are similar to those observed in (II), (III), (IV), (V), (VI) and other aryl sulfonamides (Perlovich *et al.*, 2006; Gelbrich *et al.*, 2007).

In the crystal structure,   intermolecular N–H···O hydrogen bonds (Table 1) link the molecules into inversion-related dimers. Part of the crystal structure is shown in Fig. 2.

### Experimental

The solution of toluene (10 ml) in chloroform (40 ml) was treated dropwise with chlorosulfonic acid (25 ml) at 0 ° C. After the initial evolution of hydrogen chloride subsided, the reaction mixture was brought to room temperature and poured into crushed ice in a beaker. The chloroform layer was separated, washed with cold water and allowed to evaporate slowly. The residual 4-methylbenzenesulfonylchloride was treated with 3,5-dimethylaniline in the stoichiometric ratio and boiled for ten minutes. The reaction mixture was then cooled to room temperature and added to ice cold water (100 ml). The resultant *N*-(3,5-dimethylphenyl)4-methylbenzenesulfonamide was filtered under suction and washed thoroughly with cold water. It was then recrystallized to constant melting point from dilute ethanol. The purity of the compound was checked and characterized by recording its infrared and NMR spectra (Shetty & Gowda, 2005). The single crystals used in X-ray diffraction studies were grown in ethanolic solution by a slow evaporation at room temperature.

### Refinement

The H atom of the NH group was located in a difference map and later restrained to the distance N—H = 0.86 (2) Å. The other H atoms were positioned with idealized geometry using a riding model with C—H = 0.93–0.96 Å A l l H atoms were refined with isotropic displacement parameters (set to 1.2 times of the *U*_eq_ of the parent atom).

### Crystal data

**Table:**

C_15_H_17_NO_2_S	*Z* = 2
*M**_r_* = 275.36	*F*(000) = 292
Triclinic, *P*1	*D*_x_ = 1.233 Mg m^−^^3^
Hall symbol: -P 1	Mo *K*α radiation, λ = 0.71073 Å
*a* = 8.311 (1) Å	Cell parameters from 2279 reflections
*b* = 8.521 (1) Å	θ = 2.6–27.7°
*c* = 11.412 (1) Å	µ = 0.22 mm^−^^1^
α = 99.86 (1)°	*T* = 299 K
β = 97.62 (1)°	Prism, colourless
γ = 108.14 (1)°	0.45 × 0.45 × 0.40 mm
*V* = 741.60 (14) Å^3^	

### Data collection

**Table:**

Oxford Diffraction Xcalibur diffractometer with a Sapphire CCD detector	3025 independent reflections
Radiation source: fine-focus sealed tube	2525 reflections with *I* > 2σ(*I*)
graphite	*R*_int_ = 0.012
Rotation method data acquisition using ω and phi scans	θ_max_ = 26.4°, θ_min_ = 2.6°
Absorption correction: multi-scan (*CrysAlis RED*; Oxford Diffraction, 2009)	*h* = −10→9
*T*_min_ = 0.909, *T*_max_ = 0.919	*k* = −10→10
4897 measured reflections	*l* = −14→13

### Refinement

**Table:**

Refinement on *F*^2^	Secondary atom site location: difference Fourier map
Least-squares matrix: full	Hydrogen site location: inferred from neighbouring sites
*R*[*F*^2^ > 2σ(*F*^2^)] = 0.040	H atoms treated by a mixture of independent and constrained refinement
*wR*(*F*^2^) = 0.118	*w* = 1/[σ^2^(*F*_o_^2^) + (0.0643*P*)^2^ + 0.1701*P*] where *P* = (*F*_o_^2^ + 2*F*_c_^2^)/3
*S* = 1.06	(Δ/σ)_max_ = 0.025
3025 reflections	Δρ_max_ = 0.27 e Å^−^^3^
179 parameters	Δρ_min_ = −0.31 e Å^−^^3^
1 restraint	Extinction correction: *SHELXL97* (Sheldrick, 2008), Fc^*^=kFc[1+0.001xFc^2^λ^3^/sin(2θ)]^-1/4^
Primary atom site location: structure-invariant direct methods	Extinction coefficient: 0.087 (7)

### Special details

**Table:**

Experimental. CrysAlis RED (Oxford Diffraction, 2009) Empirical absorption correction using spherical harmonics, implemented in SCALE3 ABSPACK scaling algorithm.
Geometry. All e.s.d.'s (except the e.s.d. in the dihedral angle between two l.s. planes) are estimated using the full covariance matrix. The cell e.s.d.'s are taken into account individually in the estimation of e.s.d.'s in distances, angles and torsion angles; correlations between e.s.d.'s in cell parameters are only used when they are defined by crystal symmetry. An approximate (isotropic) treatment of cell e.s.d.'s is used for estimating e.s.d.'s involving l.s. planes.
Refinement. Refinement of *F*^2^ against ALL reflections. The weighted *R*-factor *wR* and goodness of fit *S* are based on *F*^2^, conventional *R*-factors *R* are based on *F*, with *F* set to zero for negative *F*^2^. The threshold expression of *F*^2^ > σ(*F*^2^) is used only for calculating *R*-factors(gt) *etc*. and is not relevant to the choice of reflections for refinement. *R*-factors based on *F*^2^ are statistically about twice as large as those based on *F*, and *R*- factors based on ALL data will be even larger.

### Fractional atomic coordinates and isotropic or equivalent isotropic displacement parameters (Å^2^)

**Table:**

	*x*	*y*	*z*	*U*_iso_*/*U*_eq_	
S1	0.81313 (5)	1.06718 (5)	0.37422 (4)	0.04434 (17)	
O1	0.91624 (16)	1.14988 (15)	0.49312 (11)	0.0528 (3)	
O2	0.76542 (18)	1.16623 (17)	0.29675 (13)	0.0587 (4)	
N1	0.92841 (19)	0.9671 (2)	0.30816 (13)	0.0485 (4)	
H1N	0.977 (3)	0.928 (3)	0.3581 (17)	0.058*	
C1	0.6248 (2)	0.9101 (2)	0.38708 (15)	0.0413 (4)	
C2	0.4799 (2)	0.8575 (3)	0.29635 (18)	0.0550 (5)	
H2	0.4794	0.9097	0.2312	0.066*	
C3	0.3358 (2)	0.7272 (3)	0.3029 (2)	0.0612 (5)	
H3	0.2378	0.6926	0.2418	0.073*	
C4	0.3338 (2)	0.6468 (2)	0.3982 (2)	0.0565 (5)	
C5	0.4797 (3)	0.7038 (3)	0.4893 (2)	0.0617 (5)	
H5	0.4794	0.6531	0.5552	0.074*	
C6	0.6256 (2)	0.8341 (2)	0.48470 (17)	0.0541 (5)	
H6	0.7230	0.8701	0.5464	0.065*	
C7	0.8582 (2)	0.8517 (2)	0.19231 (15)	0.0457 (4)	
C8	0.8165 (3)	0.9108 (3)	0.09055 (17)	0.0566 (5)	
H8	0.8323	1.0252	0.0976	0.068*	
C9	0.7514 (3)	0.7991 (3)	−0.02150 (18)	0.0640 (5)	
C10	0.7315 (3)	0.6301 (3)	−0.03010 (19)	0.0695 (6)	
H10	0.6872	0.5549	−0.1054	0.083*	
C11	0.7759 (3)	0.5689 (3)	0.07052 (19)	0.0629 (5)	
C12	0.8382 (2)	0.6827 (3)	0.18233 (17)	0.0530 (4)	
H12	0.8668	0.6447	0.2512	0.064*	
C13	0.1768 (3)	0.5018 (3)	0.4026 (3)	0.0824 (8)	
H13A	0.0804	0.5406	0.4061	0.099*	
H13B	0.1993	0.4592	0.4734	0.099*	
H13C	0.1505	0.4131	0.3313	0.099*	
C14	0.7039 (4)	0.8623 (4)	−0.1327 (2)	0.0946 (9)	
H14A	0.5812	0.8369	−0.1510	0.113*	
H14B	0.7392	0.8075	−0.2003	0.113*	
H14C	0.7613	0.9827	−0.1174	0.113*	
C15	0.7573 (4)	0.3850 (3)	0.0585 (3)	0.0952 (9)	
H15A	0.6505	0.3153	0.0046	0.114*	
H15B	0.7577	0.3579	0.1368	0.114*	
H15C	0.8520	0.3645	0.0266	0.114*	

### Atomic displacement parameters (Å^2^)

**Table:**

	*U*^11^	*U*^22^	*U*^33^	*U*^12^	*U*^13^	*U*^23^
S1	0.0445 (3)	0.0386 (2)	0.0471 (3)	0.00888 (17)	0.00667 (18)	0.01407 (18)
O1	0.0526 (7)	0.0424 (7)	0.0528 (7)	0.0073 (5)	0.0022 (6)	0.0067 (6)
O2	0.0648 (8)	0.0499 (7)	0.0652 (8)	0.0178 (6)	0.0106 (7)	0.0280 (7)
N1	0.0437 (8)	0.0558 (9)	0.0448 (8)	0.0144 (7)	0.0068 (6)	0.0145 (7)
C1	0.0405 (8)	0.0376 (8)	0.0448 (9)	0.0119 (7)	0.0082 (7)	0.0093 (7)
C2	0.0479 (10)	0.0609 (12)	0.0535 (11)	0.0149 (9)	0.0042 (8)	0.0176 (9)
C3	0.0420 (10)	0.0599 (12)	0.0691 (13)	0.0089 (8)	0.0020 (9)	0.0045 (10)
C4	0.0468 (10)	0.0402 (9)	0.0802 (14)	0.0109 (8)	0.0223 (9)	0.0072 (9)
C5	0.0647 (12)	0.0540 (11)	0.0706 (13)	0.0150 (9)	0.0225 (10)	0.0281 (10)
C6	0.0514 (10)	0.0519 (10)	0.0546 (11)	0.0096 (8)	0.0049 (8)	0.0203 (9)
C7	0.0406 (9)	0.0540 (10)	0.0431 (9)	0.0136 (7)	0.0111 (7)	0.0153 (8)
C8	0.0644 (12)	0.0592 (12)	0.0521 (11)	0.0235 (10)	0.0135 (9)	0.0224 (9)
C9	0.0738 (14)	0.0770 (14)	0.0473 (11)	0.0312 (11)	0.0081 (9)	0.0224 (10)
C10	0.0846 (15)	0.0734 (14)	0.0468 (11)	0.0286 (12)	0.0047 (10)	0.0075 (10)
C11	0.0757 (14)	0.0589 (12)	0.0533 (11)	0.0238 (10)	0.0093 (10)	0.0123 (9)
C12	0.0574 (11)	0.0605 (11)	0.0465 (10)	0.0229 (9)	0.0109 (8)	0.0207 (9)
C13	0.0585 (13)	0.0538 (12)	0.130 (2)	0.0071 (10)	0.0356 (14)	0.0180 (14)
C14	0.126 (2)	0.113 (2)	0.0554 (14)	0.0512 (19)	0.0057 (14)	0.0353 (14)
C15	0.140 (3)	0.0633 (15)	0.0771 (17)	0.0353 (16)	0.0095 (17)	0.0107 (13)

### Geometric parameters (Å, °)

**Table:**

S1—O2	1.4220 (13)	C8—C9	1.383 (3)
S1—O1	1.4353 (13)	C8—H8	0.9300
S1—N1	1.6414 (16)	C9—C10	1.382 (3)
S1—C1	1.7570 (17)	C9—C14	1.513 (3)
N1—C7	1.431 (2)	C10—C11	1.393 (3)
N1—H1N	0.830 (15)	C10—H10	0.9300
C1—C2	1.378 (2)	C11—C12	1.388 (3)
C1—C6	1.381 (2)	C11—C15	1.506 (3)
C2—C3	1.378 (3)	C12—H12	0.9300
C2—H2	0.9300	C13—H13A	0.9600
C3—C4	1.381 (3)	C13—H13B	0.9600
C3—H3	0.9300	C13—H13C	0.9600
C4—C5	1.382 (3)	C14—H14A	0.9600
C4—C13	1.506 (3)	C14—H14B	0.9600
C5—C6	1.380 (3)	C14—H14C	0.9600
C5—H5	0.9300	C15—H15A	0.9600
C6—H6	0.9300	C15—H15B	0.9600
C7—C12	1.380 (3)	C15—H15C	0.9600
C7—C8	1.387 (2)		
			
O2—S1—O1	119.35 (8)	C7—C8—H8	120.1
O2—S1—N1	108.43 (8)	C10—C9—C8	119.03 (19)
O1—S1—N1	104.34 (8)	C10—C9—C14	120.8 (2)
O2—S1—C1	108.50 (8)	C8—C9—C14	120.1 (2)
O1—S1—C1	109.14 (8)	C9—C10—C11	122.0 (2)
N1—S1—C1	106.34 (8)	C9—C10—H10	119.0
C7—N1—S1	120.83 (12)	C11—C10—H10	119.0
C7—N1—H1N	112.8 (15)	C12—C11—C10	117.9 (2)
S1—N1—H1N	109.6 (15)	C12—C11—C15	121.0 (2)
C2—C1—C6	120.44 (16)	C10—C11—C15	121.1 (2)
C2—C1—S1	119.58 (13)	C7—C12—C11	120.66 (17)
C6—C1—S1	119.90 (13)	C7—C12—H12	119.7
C3—C2—C1	119.53 (18)	C11—C12—H12	119.7
C3—C2—H2	120.2	C4—C13—H13A	109.5
C1—C2—H2	120.2	C4—C13—H13B	109.5
C2—C3—C4	121.32 (18)	H13A—C13—H13B	109.5
C2—C3—H3	119.3	C4—C13—H13C	109.5
C4—C3—H3	119.3	H13A—C13—H13C	109.5
C5—C4—C3	118.06 (17)	H13B—C13—H13C	109.5
C5—C4—C13	121.2 (2)	C9—C14—H14A	109.5
C3—C4—C13	120.7 (2)	C9—C14—H14B	109.5
C4—C5—C6	121.66 (19)	H14A—C14—H14B	109.5
C4—C5—H5	119.2	C9—C14—H14C	109.5
C6—C5—H5	119.2	H14A—C14—H14C	109.5
C5—C6—C1	118.96 (18)	H14B—C14—H14C	109.5
C5—C6—H6	120.5	C11—C15—H15A	109.5
C1—C6—H6	120.5	C11—C15—H15B	109.5
C12—C7—C8	120.51 (18)	H15A—C15—H15B	109.5
C12—C7—N1	119.34 (16)	C11—C15—H15C	109.5
C8—C7—N1	120.11 (17)	H15A—C15—H15C	109.5
C9—C8—C7	119.83 (19)	H15B—C15—H15C	109.5
C9—C8—H8	120.1		
			
O2—S1—N1—C7	−59.70 (15)	C2—C1—C6—C5	0.6 (3)
O1—S1—N1—C7	172.11 (13)	S1—C1—C6—C5	−176.08 (15)
C1—S1—N1—C7	56.80 (15)	S1—N1—C7—C12	−116.91 (17)
O2—S1—C1—C2	24.58 (17)	S1—N1—C7—C8	65.4 (2)
O1—S1—C1—C2	156.11 (15)	C12—C7—C8—C9	1.2 (3)
N1—S1—C1—C2	−91.87 (16)	N1—C7—C8—C9	178.92 (17)
O2—S1—C1—C6	−158.72 (15)	C7—C8—C9—C10	−1.0 (3)
O1—S1—C1—C6	−27.19 (17)	C7—C8—C9—C14	179.4 (2)
N1—S1—C1—C6	84.83 (16)	C8—C9—C10—C11	−0.3 (4)
C6—C1—C2—C3	−0.6 (3)	C14—C9—C10—C11	179.3 (2)
S1—C1—C2—C3	176.04 (15)	C9—C10—C11—C12	1.4 (4)
C1—C2—C3—C4	−0.5 (3)	C9—C10—C11—C15	−178.5 (3)
C2—C3—C4—C5	1.6 (3)	C8—C7—C12—C11	−0.1 (3)
C2—C3—C4—C13	−178.55 (19)	N1—C7—C12—C11	−177.81 (17)
C3—C4—C5—C6	−1.7 (3)	C10—C11—C12—C7	−1.2 (3)
C13—C4—C5—C6	178.5 (2)	C15—C11—C12—C7	178.7 (2)
C4—C5—C6—C1	0.6 (3)		

### Hydrogen-bond geometry (Å, °)

**Table:**

*D*—H···*A*	*D*—H	H···*A*	*D*···*A*	*D*—H···*A*
N1—H1N···O1^i^	0.83 (2)	2.11 (2)	2.933 (2)	170 (2)

Symmetry codes: (i) −*x*+2, −*y*+2, −*z*+1.
